# FTRLIM: Distributed Instance Matching Framework for Large-Scale Knowledge Graph Fusion

**DOI:** 10.3390/e23050602

**Published:** 2021-05-13

**Authors:** Hongming Zhu, Xiaowen Wang, Yizhi Jiang, Hongfei Fan, Bowen Du, Qin Liu

**Affiliations:** 1 School of Software Engineering, Tongji University, Shanghai 201804, China; zhu_hongming@tongji.edu.cn (H.Z.); 2110211@tongji.edu.cn (X.W.); 1931566@tongji.edu.cn (Y.J.); fanhongfei@tongji.edu.cn (H.F.); 2 Department of Computer Science, University of Warwick, Coventry CV4 7AL, UK

**Keywords:** knowledge graph, instance matching, blocking algorithm, FTRL

## Abstract

Instance matching is a key task in knowledge graph fusion, and it is critical to improving the efficiency of instance matching, given the increasing scale of knowledge graphs. Blocking algorithms selecting candidate instance pairs for comparison is one of the effective methods to achieve the goal. In this paper, we propose a novel blocking algorithm named MultiObJ, which constructs indexes for instances based on the Ordered Joint of Multiple Objects’ features to limit the number of candidate instance pairs. Based on MultiObJ, we further propose a distributed framework named Follow-the-Regular-Leader Instance Matching (FTRLIM), which matches instances between large-scale knowledge graphs with approximately linear time complexity. FTRLIM has participated in OAEI 2019 and achieved the best matching quality with significantly efficiency. In this research, we construct three data collections based on a real-world large-scale knowledge graph. Experiment results on the constructed data collections and two real-world datasets indicate that MultiObJ and FTRLIM outperform other state-of-the-art methods.

## 1. Introduction

Knowledge graphs have the strong expressive ability and modeling flexibility as semantic networks. Many knowledge graphs have been published for a variety of practical needs, such as DBpedia [1], Freebase [2], YAGO [3], and IMDb (http://www.imdb.com, accessed on 9 December 2020). The idea of knowledge graph is widely used in intelligent question answering [4], recommendation systems [5], semantic search [6], and other fields. However, due to the lack of unified presentation standards for data and information, and/or the differences in the methods of obtaining data [7], the relevant knowledge of the same entity in the real world is represented in various forms among different knowledge graphs. It is not conducive to knowledge sharing between different domains and applications.

Instance matching (IM) is defined as establishing a specific type of semantic link between instances. The semantic link is called the identity link represented by the *owl:sameAs*. IM is also known as entity alignment [8], record linkage [9], duplicate detection [10], or co-reference resolution [11]. It allows us to explicitly link two instances that refer to the same entity in the real world. When merging different knowledge graphs, instance matching is adopted to achieve consistency and integrity.

Instance matching has attracted attentions since 2009 [12], but the realization of the ultimate solution is still an open research problem. As the scale of the built knowledge graphs increases, the efficiency and cost requirements of instance matching methods become more strict. Matching instances between knowledge graphs corresponds to the Clique problem in graph theory, which is an NP-complete problem [13,14]. The Clique problem is to find cliques in an undirected graph, where a clique is a completed subgraph. Briefly, consider two knowledge graphs to form one graph, where vertices are instances from the two knowledge graphs, and the edges are identity links. Then, a clique represents a set of instances that point to the same real-world entity. Instance matching is the problem to list all such cliques. Earlier published methods [12,15] are not suitable for processing large-scale knowledge graphs containing tens of thousands of instances, mainly because these frameworks usually require the pair-by-pair comparison among instances from different knowledge graphs. To our best knowledge, there are mainly two approaches to matching instances between large-scale knowledge graphs. (i) The blocking algorithm can be adopted to reduce the searching space. This type of approach divides instances into overlapping blocks and executes the matching process only within blocks. ServOMap [16], VMI [17], RiMOM-IM [18], ScLink [19] and other frameworks [20] leverage such approach. (ii) The distributed architecture can be utilized to provide sufficient computing resources. The distributed file system can be used to store large knowledge graphs. The distributed computing model, such as MapReduce [21], allows the instance matching process to be divided into multiple matching tasks that can be executed by multiple workers. Frameworks that adopt this include LINDA [14], BIGMAT [22], etc.

There exist challenges to be solved in the field of large-scale instance matching. (i) A large number of candidate instance pairs need to be compared during the matching process, which has an adverse impact on the matching efficiency. Although blocking algorithms can reduce the number of candidate pairs, existing blocking algorithms adopted by conventional frameworks [16,17,18] prefer to achieve high recall by replicating instances to multiple blocks. The redundancy of instances leads to the generation of extra candidate pairs, which increases the matching time. (ii) It is difficult to achieve a reasonable balance between matching efficiency and matching quality. Although standalone frameworks [18,20,23] can obtain high-quality matching results, they have high requirements for time and computing resources to match large-scale knowledge graphs. Meanwhile, several distributed frameworks [14,22] have been proposed and claimed to be able to process large-scale knowledge graphs efficiently, but their matching quality can be further improved.

To tackle the above challenges, we propose a novel blocking algorithm MultiObJ to select candidate pairs effectively. The proposed algorithm constructs inverted indexes for instances based on the ordered joint of multiple objects’ features. The results of the joint serve as evidence for blocking. Only instances from different knowledge graphs within the same block can form candidate pairs. Based on the proposed algorithm, we design a distributed instance matching framework FTRLIM (code: https://github.com/TOJSSE-iData/ftrlim, accessed on 12 May 2021). It firstly adopts MultiObJ to select candidate pairs. Then, it calculates the similarity of objects under pre-aligned predicates to generates similarity vectors for candidate pairs based on the attributes and relationships of instances. The FTRLIM framework models the problem of instance matching as logistic regression and leverages the online logistic regression model FTRL [24] to determine whether candidate pairs are matched. The framework is implemented in a distributed architecture and scales well. In addition, we construct three data collections based on real-world data with different scales and levels of heterogeneity for comprehensive evaluation. The constructed data collections can be used as benchmarks to provide a quantitative evaluation of blocking algorithms and instance matching frameworks in further researches.

FTRLIM has participated in the competition of SPIMBENCH Track at OAEI 2019 and outperformed other state-of-the-art frameworks. This paper further evaluates the MultiObJ blocking algorithm and the FTRLIM framework on the three constructed data collections and two real-world datasets. Compared with RIMOM-IM’s method [18], experiment results show that MultiObJ generates much fewer candidate pairs (1/819∼1/6 of RIMOM-IM’s) and brings a distinguished matching efficiency improvement for the FTRLIM framework. Evaluation results of matching quality show that FTRLIM achieves the same level of F1-score as the best one among more than ten advanced frameworks. Besides, FTRLIM has the capability to match instances between knowledge graphs containing more than 600,000 instances with satisfied quality and efficiency. The time cost of matching decreases as the number of available cores in the distributed cluster increases.

The main contributions of our work can be summarized as:

- We propose a novel blocking algorithm MultiObJ, which divides instances into blocks by utilizing the ordered joint of multiple objects’ features. The experiment results indicate that the proposed algorithm can significantly reduce the number of candidate instance pairs with only an inconspicuous effect on the matching quality.
- We design and implement a distributed instance matching framework FTRLIM for large-scale knowledge graphs based on MultiObJ. FTRLIM is able to match instances between large-scale knowledge graphs efficiently. It mines matched instances using the online logistic regression model follow-the-regular-leader (FTRL). The experiment results show that FTRLIM overall outperforms other state-of-the-art frameworks on real-world datasets and has excellent scalability and efficiency.
- We construct three data collections with golden-standards based on a real-world large-scale knowledge graph. Knowledge graphs in these three data collections are constructed with different scales and levels of heterogeneity to meet various evaluation purposes. We evaluate MultiObJ and FTRLIM on these three data collections and two real-world datasets. The constructed data collections and experiment results can be replicated by others and provide a potential baseline for further research.

The rest of this paper is organized as follows. In Section 2, we review related work. We formally describe the instance matching problem in Section 3. In Section 4, we describe the detailed working principle and process of the FTRLIM framework. We analyze the time complexity of our framework in Section 6. Experiments and analyses are performed in Section 5. In Section 7, we summarize this paper.

## 2. Related Work

The term knowledge graph (KG) has been widely used since Google published their work in 2012 [25]. Recently, Färber et al. use KG to describe any Resource Description Framework (RDF) graph [26]. RDF is an infrastructure that is designed for encoding, exchanging and reusing structural data [27]. It has been widely used in different domains to store and share knowledge. The European Bioinformatics Institute (EBI) develops the EBI RDF platform [28] for describing, publishing and linking life science data. The Open European Nephrology Science Center leverages the RDF model to share and search medical data among research groups [29]. The GEOLink [30] database provides geoscience metadata repositories in RDF format and allows users to perform seamlessly query and reasoning. Recently, the team of Ali develop frameworks that treat the data from social networks into structural data for traffic event detection and condition analysis [31] and for intelligent healthcare monitoring [32].

Although RDF is a standard language for describing resources on the network, the description could be subjective and be various in different applications, which creates obstacles to knowledge sharing in the same domain or even across domains. One of the ways to overcome the obstacle is instance matching. Many methods have been proposed to complete the instance matching task. Several state-of-the-art instance matching methods evolve from ontology matching methods, such as LogMap [33], AML [34], RiMOM-IM [18], and Lily [35]. The first three frameworks adopt the idea of bootstrapping and iteratively discover more matched instance pairs based on pairs that are already matched. PARIS [23] adopts a similar idea and models the probability that two instances can match. It is able to match both schema and instances. Lily [35] focuses more on ontology matching and manual adjustments are required when completing the instance matching task. VMI [17] and VDLS [20] model the instance matching problem as a document matching problem and build vectors for instances based both on their local information and their neighbors’ information. They determine whether two instances are matched by calculating the similarity between their vectors. SERIMI [36] selects the most discriminative attribute of instances by computing the entropy of each attribute and builds the pseudo-homonyms sets of instances. They complete the class-based disambiguation of instances by their set similarity function.

Researchers have been exploring applying machine learning and deep learning methods to the solution of instance matching problems. Supervised learning-based methods [37,38,39] have been applied in instance matching problem, which consider instance matching a binary classification problem. These methods require labeling instances to train the model. Among them, TrAdaBoost [38] adopts the transfer learning algorithm to obtain training data, which reduces the manual work of labeling. Moreover, rather than training models to match instances, MDedup [40] trains models for discovering the matching dependencies (MDs) to select matched instances, where MD is one of the relaxed forms [41,42] of functional dependency [43] in data mining. Semi-supervised learning methods [44,45], unsupervised learning methods [46,47] and self-supervised learning model [48] are also introduced into the field of instance matching. Besides, works on representation learning for matching instances are gradually emerging [49,50,51]. These methods firstly embed instances in each graph into different low-dimensional dense semantic spaces separately. Then, they align the spaces according to the pre-matched instances to find more matched instance pairs. There are also frameworks designed for training and evaluating the embedding models, such as References [52,53,54]. Compared with other works, the FTRL model is more lightweight, and it can give the probability that two instances are matched. We introduce FTRL in more detail in Section 4.3.

How to deal with large-scale data has become an inevitable problem in instance matching. As described in Section 1, the instance matching problem corresponds to the NP-complete Clique problem. The most popular solution for large-scale IM is blocking. This approach divides similar instances into blocks and limits the comparison within blocks. There are views that blocking-based instance matching is the best approach for efficient matching [55]. Some blocking algorithms require manual works [56]. Moreover, automated blocking algorithms are applied by different instance matching frameworks [16,17,18,34]. These methods generate inverted indexes for instances by analyzing their attributes or types. Blocks are generated according to these indexes. The blocking approach can split the large-scale instance matching task into multiple subtasks. Therefore, it is usually performed as the first step of large-scale instance matching methods. A more detailed survey is presented in Papadakis’s research [57]. The most similar blocking method to us may be the one proposed in Reference [18]. This method distinguishes the objects related to different predicates and regards the instance pair with a unique index, i.e., the unique pair, as a matched pair. The obvious difference is that we also consider the correlation among different predicates, which further reduces the overlap between blocks. We use the features of the object rather than always using the entire object to construct block keys, which improves the robustness. Moreover, we only consider unique pairs as a special type of candidate pairs, rather than directly as matched pairs, to improve the precision.

Adopting the distributed architecture is another way to perform large-scale instance matching. The LINDA framework [14] performs instance matching by considering joint evidence for instances and adopts a distributed version of the algorithm. MSBlockSlicer [58] pays attention to the problem of load imbalance and adopts a block slice strategy to balance the load of each worker in the distributed cluster. The BIGMAT framework [22] applies the affinity-preserving random walk algorithm to express IM as a graph-based node ranking and selection problem in the constructed candidate association graph and selects matching results through a distributed architecture. Our framework leverages the proposed blocking algorithm to divide the matching task into multiple logistic regression tasks that can be executed distributionally. We also introduce the load balancing mechanism to make full use of cluster resources.

As the number of proposed methods increases, researchers construct the Ontology Alignment Evaluation Initiative (OAEI) to evaluate these methods. The evaluation is carried out based on multiple tracks. The SPIMBENCH Track is one of the newest tracks for instance matching evaluation. FTRLIM was evaluated on this track in 2019 and out-performed other state-of-the-art frameworks.

## 3. Problem Formulation

### 3.1. Knowledge Graph

A knowledge graph is a finite set of pieces of knowledge presented as RDF triples. An RDF triple is described in the form of $\langle s,p,o\rangle$, where s,p,o represent subject, predicate, and object, respectively. A subject is a certain instance. A predicate specifies an attribute of the subject when the object is literal text, while it defines a relationship between the subject and the object if the object is an instance. Let the instance set be *I*, the predicate set be *P*, and the literal set be *L*, and the knowledge graph is defined as

$$KG=\{\langle s,p,o\rangle |s\in I,p\in P,o\in I\cup L\}.$$

There are two types of *p* in the $\langle s,p,o\rangle$ triple. Let $O_{p}$ denote the set of *o* who makes $\langle *,p,o\rangle$ a valid triple, where ∗ is a certain instance. When $O_{p}$ is a finite set, *p* is an enumerative predicate; when it is an infinite set, *p* is a diverse predicate. The predicate type determines the strategy of constructing indexes for the instances, which will be introduced in Section 4.1.

### 3.2. Instance Matching

We use capital letters *S* and *T* as subscripts to indicate the data source, where *S* for source KG and *T* for target KG. It is the same as the succeeding text. When given the source knowledge graph $KG_{S}$ and the target knowledge graph $KG_{T}$, instance matching task requires to identify all instance pairs $\langle i,j\rangle$ that satisfy $\langle i,\mathrm{owl}:\mathrm{sameAs},j\rangle$, where $i\in I_{S},j\in I_{T}$. A pair of instances that meet the condition is called to be matched. In this paper, we believe that the matching process follows two assumptions.

**Assumption** **A1.** $P_{S}\cap P_{T}\neq \emptyset$*.*

This assumption can be interpreted from two perspectives: (1) The source knowledge graph and the target knowledge graph have predicates describing the same attribute or relationship of the instances. (2) These predicates are aligned, which means the description of the same aspect of the instances in different KGs is given through exactly the same predicate. The method of aligning predicates has been widely studied in the field of ontology matching since 2003 [59]. We believe that this assumption can be satisfied in the field of instance matching.

**Assumption** **A2.** *In the target knowledge graph, at most, one instance matches the instance in the source knowledge graph, and vice versa.*

Our work focuses on matching instances between non-homologous KGs. It means that instances in the same knowledge graph should be different from each other.

FTRLIM regards IM as a logistic regression problem, where the regression values are the similarity scores of instance pairs. The function that indicates the similarity between instance $i\in I_{S}$ and $j\in I_{T}$ is defined as $Sim(i,j,KG_{S},KG_{T})$, in which the value range is [0,1]. The larger the similarity is, the more likely the two instances will be matched. A formal description of instance matching is defined as follows. For instances $i\in I_{S},j\in I_{T}$, *i* and *j* are matched if and only if:

(1) $$\left\{\begin{array}{c} Sim\left(i,j,KG_{S},KG_{T}\right)\geq Sim\left(i,k,KG_{S},KG_{T}\right),\forall k\in I_{T}\\ Sim\left(i,j,KG_{S},KG_{T}\right)\geq Sim\left(k,j,KG_{S},KG_{T}\right),\forall k\in I_{S}\\ Sim(i,j,KG_{S},KG_{T})\geq \theta \end{array},\right.$$

where θ is a manually set threshold.

## 4. The FTRLIM Framework

**Figure 1:**
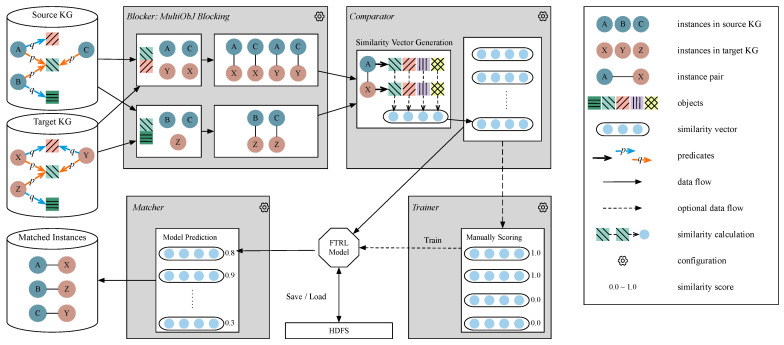
Workflow of the proposed FTRLIM framework for large-scale instance matching.

This section introduces the detailed working process of FTRLIM. The proposed framework consists of four major components: *Blocker*, *Comparator*, *FTRL Trainer*, and *Matcher*. The overview of the FTRLIM’s workflow is presented in Figure 1. *Blocker* obtains instance pairs to be compared, which adopts the proposed MultiObJ blocking algorithm to reduce the number of candidate pairs. *Comparator* is responsible for generating similarity vectors for each instance pair. *FTRL Trainer* takes similarity vectors and their scores as inputs to train the FTRL model, while *Matcher* adopts the trained model to determine whether instances are matched. The training process is optional because FTRLIM allows users to load a pre-trained model. The framework is implemented in a distributed architecture.

### 4.1. Blocker

Identifying matched instance pairs by performing comparisons between every two instances is time and space-consuming. To solve this problem, FTRLIM adopts the **MultiObJ** blocking algorithm to efficiently select candidate instance pairs that are more likely to be matched. This work is done by *Blocker*.

The basic idea of the MultiObJ blocking algorithm is to construct indexes for each instance by leveraging features of the related objects. When constructing indexes, the interactions among different predicates of the instance should also be considered. Features of the objects under different predicates should be jointed to form the indexes of the instance, which allows instances to be fine-grained divided. This idea is intuitive: In the real world, researchers can use multiple attributives when describing an instance. The more attributives there are, the easier it is for others to locate the instance.

The MultiObJ blocking algorithm accepts triples of knowledge from both source KG and target KG and a predefined list of predicates *P* as the inputs, and it gives candidate instance pairs and unique instance pairs as the outputs. It includes three phases: *Initialization*, *Indexing*, and *Candidate Pair Generation*. In the *Initialization* phase, MultiObJ first creates the candidate pair set *C*, the unique pair set *U*, the index table *K*, and the inverted index table *B*. The index table *K* is prepared for storing indexes of instances, while the inverted index table *B* is prepared for storing the mapping from a certain index to instances. Then, it allocates a common initial inverted index $k_{init}$ for all instances that belong to either source or target KG and updates *K* and *B*, which leads to all instances are under the same block at the very beginning. In the *Indexing* phase, the algorithm sequentially extracts the features of related objects according to the predefined predicate list *P* and constructs inverted indexes for the instances. The instances with the same index are divided into the same block. As the iteration deepens, the large block will be subdivided into multiple small blocks. The processing of instances in which the objects are missing is also supported. The *Candidate Pair Generation* phase is responsible for combining instances from different knowledge graphs in the same block into candidate instance pairs.

The core phase of the algorithm is the *Indexing* phase, which includes three subphases: *Explicit Indexing*, *Unique Pair Generation*, and *Index Inference*. The algorithm processes each predicate *p* in the predefined predicate list *P* iteratively. Each iteration owns two additional predicate-specified tables: the indexing table $K_{p}$ and the inverted indexing table $B_{p}$. These two tables are used for storing provisional results that are passed to *K* and *B* at the end of each iteration. The MultiObJ algorithm aims at leveraging object features of instances to divide blocks. It extracts object features, builds inverted indexes for instances in the *Explicit Indexing* phase, and utilizes the unique information among these features to generate unique pairs in the *Unique Pair Generation* phase. However, instances may have no corresponding object under certain predicates. MultiObJ infers possible features of these instances in the *Index Inference* phase. The following paragraphs will introduce the details of the algorithm.

We name the initial index and the indexes generated for the instance *i* in the previous iteration as the pre-index of the instance *i*. The object set of *i* under the predicate *p* is noted as i[p]. In the *Explicit Indexing* phase, all instances are divided into two parts depending on whether i[p] exists with the function *devideByObjectMissing*. This phase concentrates on building indexes for instances with i[p]. In this phase, the features of the object *o* are extracted with the function *extractObjectFeatureSet*, where o∈i[p]. The strategies of feature extraction will be introduced later. The algorithm concatenates the extracted features with each pre-index of the instance *i* as the current indexes using the function *catPreIdxAndFeature*, and records the result in $K_{p}$. The inverted index table $B_{p}$ is also updated using the function *updateInvertedIndexTable*.

In the *Unique Pair Generation* phase, the algorithm aims to detect the unique instance pairs. If and only if there is one pair of instances from different data sources with a certain index, these two instances are considered a unique instance pair. When two instances have the same index, and the index is unique in the source KG and the target KG, they are the most likely to be matched intuitively. FTRLIM achieves this intuition by setting a lower threshold for the unique pairs when determining whether two instances are matched, which will be introduced later.

The lack of knowledge is considered in the *Index Inference* phase to avoid losing candidate instance pairs as much as possible. If the expected object of instance *s* from the source KG under the predicate *p* is missing, it means that the lack of knowledge occurs in the source KG. MultiObJ will identify all the instances in the target KG that have the same pre-index as *s* using the function *getInstByPreIdx* and use all their indexes generated according to *p* as the index of *s*. Moreover, *s* is also indexed by a special string *NULL* to indicate that it has no corresponding object under the predicate *p*. The same process is performed on instances with missing objects in target KG. In this way, the instances without object under *p* will have a wildcard as an index, so they can still form candidate pairs with other instances. When an iteration ends, the current indexes for instances become the new pre-indexes. The pseudo-code of the MultiObJ blocking algorithm are shown in Algorithm 1.
**Algorithm 1** MultiObJ**Input:** *S*  source knowledge graph *T*  target knowledge graph *P*  list of predicates used to generate indexes **Output:** *C*  candidate pair set *U*  unique pair set ***Initialization***1:U←∅,C←∅,B←∅,K←∅2:**for all** i∈S∪T **do**3: $K\left[i\right].add\left(k_{init}\right)$4: $B\left[k_{init}\right]\left[i.kg\right].add\left(i\right)$***Indexing***5:**for all** p∈P **do**6: $K_{p}\leftarrow \emptyset$ $B_{p}\leftarrow \emptyset$ ***Explicit Indexing***7: **for all** G∈{S,T} **do**8:  $G_{v},G_{n}\leftarrow devideByObjectMissing\left(G,p\right)$9:  **for all** $i\in G_{v}$ **do**10:   $F_{p,i}\leftarrow extractObjFeatureSet\left(i\left[p\right]\right)$11:   $K_{p}\left[i\right]\leftarrow catPreIdxAndFeature(K\left[i\right],F_{p,i}$)12:   $updateInvertedIndexTable(B_{p},K_{p}\left[i\right])$ ***Unique Pair Generation***13: **for all** $k\in B_{p}.keys\left(\right)$ **do**14:  **if** $B_{p}\left[k\right]\left[S\right].size\left(\right)==B_{p}\left[k\right]\left[T\right].size\left(\right)==1$ **then**15:   $U.add(\left(B_{p}\left[k\right]\left[S\right].get\left(\right),B_{p}\left[k\right]\left[T\right].get\left(\right)\right))$ ***Index Inference***16: **for all** $G_{n},G_{v}^{\prime }\in \left\{\left(S_{n},T_{v}\right),\left(T_{n},S_{v}\right)\right\}$ **do**17:  **for all** $i\in G_{n}$ **do**18:   $K_{n}\leftarrow catPreIdxAndFeature\left(K\left[i\right],NULL\right)$19:   **for all** $j\in getInstByPreIdx(G_{v}^{\prime },K\left[i\right])$ **do**20:    $k_{v}\leftarrow catPreIdxAndFeature\left(K\left[i\right],K_{p}\left[j\right]\right)$21:    $K_{n}.update\left(k_{v}\right)$22:    $updateInvertedIndexTable(B_{p},K_{p}\left[i\right])$23:   $K_{p}\left[i\right]\leftarrow K_{n}$24: $K\leftarrow K_{p},B\leftarrow B_{p}$***Candidate Pair Generation***25:**for all** k∈B.keys() **do**26: **for all** i∈B[k][S]×B[k][T] **do**27:  C.add(i,j)28:**return** C,U


An example of the *Indexing* phase is given in Table 1 and Table 2. Table 1 shows the relationship between the object features, the current indexes and the pre-indexes of each instance in an iteration. The current indexes of S3 and T3 are generated in the *Index Inference* phase due to the lack of related objects. Table 2 shows the blocking results in this case. It should be pointed out that $\langle S4,\;T4\rangle$ is a unique pair, while $\langle S2,\;T3\rangle$ and $\langle S3,\;T3\rangle$ are not. It is because the unique pair generation is completed before the *Index Inference* phase. Such a setting can reflect that the inferred indexes are not so reliable as the directly constructed indexes.

The 10th line of MultiObJ requires to extract objects’ features to construct instance indexes with the function *extractObjFeatureSet*. Many methods have been proposed to implement this function, such as extracting keywords with TF-IDF, extracting tokens with q-grams, and use the first three to four letters as tokens [60]. We believe that different feature extraction methods and indexing strategies should be adopted for texts with different lengths and types. During our exploration of data, we have observed that the objects corresponding to some predicates are always in a finite set, while others are not. Specifically, we divide predicates into two types, the enumerative predicate (*EP*) and the diverse predicate (*DP*). Objects of *EP* can form an enumerated set, while objects of *DP* are variable with subjects. Considering about the subject of type *people*, predicate *hasGender* is an enumerative predicate, while predicate *hasName* is a diverse predicate. Therefore, there are two index construction strategies that can be applied. For enumerative predicates, features of their corresponding objects are the objects themselves, which can be adopted as the construction basis of instance indexes after the unified processing. This construction strategy is called full index construction (*FIC*) strategy. For diverse predicates, keywords of their corresponding objects can be extracted to form the features for constructing indexes. This is the keyword index construction (*KIC*) strategy. Since *EP* is more reliable than *DP*, applying the *FIC* strategy before the *KIC* strategy will reduce the chance of the instance being incorrectly blocked in MultiObJ.

For the *KIC* strategy, we also design a new algorithm **CombKey** to deal with the long text. It extracts more discriminative features of objects to generate blocks with lower overlap. The algorithm first densely ranks the words in objects according to the word frequency from low to high. Words with higher rank are considered as keywords. After that, CombKey combines keywords in pairs as tokens according to the ranking. Since the possibility of repeated words within an object is low, only word frequency is used as the ranking indicator when considering the cost of calculating TF-IDF and other complex indicators. The detail of the CombKey algorithm is shown in Algorithm 2. The CombKey algorithm is designed for text in which the length is larger than a threshold, where the threshold is 2 empirically. For shorter texts, each word can be used as an object feature to improve robustness.
**Algorithm 2** CombKey**Input:** *i*[*p*]  object of instance *i* under predicate *p* *C_p_* word frequency counter of objects under predicate *p* *R*   maximum rank of words used for extracting features **Output:** $K_{p}$   set of object features of instance *i* under predicate *p*1:$K_{p}\leftarrow \emptyset$2:$W_{p}\leftarrow split\left(i\left[p\right]\right)$3:$R_{i}\leftarrow DenseRank\left(W_{p},C_{p},R\right)$4:**if** $R_{i}.keys\left(\right).size\left(\right)==1$ **then**5: **for all** $w\in R_{i}\left[1\right]$ **do**6:  $K_{p}.add\left(w\right)$7:**else**8: **for** $r\leftarrow 1,min(R-1,R_{i}.keys\left(\right).size\left(\right)-1)$ **do**9:  **for all** $w\in R_{i}\left[r\right]$ **do**10:   **for** $j\leftarrow r+1,min(R,R_{i}.keys\left(\right).size\left(\right))$ **do**11:    **for all** $ww\in R_{i}\left[j\right]$ **do**12:     $K_{p}.add\left(concat\left(w,\;ww\right)\right)$13:**return** $K_{p}$

Table 3 demonstrates an example of CombKey results on the Restaurant dataset with R=2. The format of the ID is *KG-Instance*. In CombKey, the names of the given instances are split into words and counted globally. Then, CombKey densely ranks the words referring to their frequency. In the end, CombKey combines words with different ranks as objects’ features. Although the word ’club’ occurs in all instances’ names, CombKey avoids regarding the single word as a feature and distinguishes the first two instances from the last two instances.

Another example is given in Figure 1 to illustrate how the MultiObJ blocking algorithm works. The algorithm leverages the objects’ features under the predicates *p* (the orange arrow) and *q* (the blue arrow) in turn to construct indexes for the instances. The six instances in Figure 1 have the same object under *p* so that they will be divided into the same block first. Then, according to the object under *q*, instances A,X, and *Y* will be divided into one new block, while instances *B* and *Z* will be divided into another block. Note that instance *C* has no object under *q*. MultiObJ will check the indexes of instances X,Y, and *Z* as part of the inference results of *C*’s indexes. This is because the three instances are in the same block divided according to objects under *p* as *C* but are from the target KG. In this case, *C* will be divided into both the block contains *X* and *Y*, and the block contains *Z*.

In a knowledge graph, if the number of instances with a certain index is much greater than the number of instances with another one, the problem of data skew will occur and affect the efficiency of subsequent calculations. We introduce the load balance mechanism to avoid the problem of data skew. FTRLIM draws the FastAGMS draft [61] for the distribution of indexes of instances, then estimates the workload of cores in the cluster and reassigns the work to balance the load.

### 4.2. Comparator

To obtain the similarity of the pair of instances, all candidate pairs are sent to the *Comparator*. The *Comparator* compares two instances under various predicates in different ways. The edit distance similarity is calculated for textual instance attributes, while the overlap similarity or the Jaccard similarity is calculated for instance relations. The calculation results will be sorted in order to form the similarity vector. Formally, let the list of predicates adopted by *Comparator* be $\langle p_{1},p_{2},\cdots ,p_{n}\rangle$, then the similarity vector of the two instances is

$$\langle s_{1},s_{2},\cdots ,s_{n}\rangle ,s_{i}\in \left[0,1\right],\left(i=1,2,\cdots ,n\right),$$

where $s_{i}$ is the similarity of the two instances under the *i*-th predicate. Table 4 shows an example of similarity vector generation. The listed instances are two documents. The column Sim1 represents the edit distance similarity of their labels, and the column Sim2 represents the overlap similarity of sets of their authors.

When calculating the similarity of instance pairs under a certain predicate, some instances may have no corresponding objects due to the data flaws of the knowledge graph itself. A naïve way to obtain the similarity is to assign it a default value 0. However, this solution may confuse the difference between *the lack of knowledge* and *the dissimilarity*. To differentiate the two cases more clearly, we use the ratio of the number of instances with objects under a predicate to the total number of instances to represent the completeness rate of this predicate. If most instances have objects under a predicate, an instance may be more distinctive when its object is missing. Based on this consideration, we believe that the higher the predicate’s completeness, the lower the similarity between the instance without objects and other instances should be. Formally, we define the default similarity $Sim_{d}$ for instance pairs without attributes or relations as:

(2) $$Sim_{d}=1-\frac{1}{2}\left(\frac{|I_{S,p}|}{|I_{S}|}+\frac{|I_{T,p}|}{|I_{T}|}\right),$$

where $I_{S},I_{T}$ indicate the instance sets of source and target knowledge graphs, and $I_{S,p},I_{T,p}$ are the sets of instances with objects corresponding to the predicate *p*. The term $\frac{|I_{*,p}|}{|I_{*}|}$ is the completeness of the predicate *p* in the source or target KG.

### 4.3. FTRL Trainer

As described in Section 3.2, FTRLIM treats IM as a logistic regression problem. We innovatively introduce the FTRL model [24] to solve the problem. FTRL is an advanced online logistic regression model with high precision and excellent sparsity. It is designed to apply the logistic regression on large-scale datasets and online data streaming, which is a difficult situation for the conventional batch learning model. FTRL also has a fast training speed. Hence, we choose FTRL to discover matched instance pairs.

Let $\vec{x}$ be the similarity vector, and *y* be the label of $\vec{x}$, the FTRL model gives the predicted label $\hat{y}$ of $\vec{x}$ with the sigmoid function:

(3) $$\hat{y}=\frac{1}{1+\exp(-{\vec{w}}^{T}\cdot \vec{x})},$$

where $\vec{w}$ is the weight vector of the FTRL model.

The loss function of the FTRL model is the binary cross-entropy loss, which is defined as:

(4) $$\ell =-(y\log\hat{y}+\left(1-y\right)\log\left(1-\hat{y}\right)).$$

The formula of updating the FTRL model’s weight $\vec{w}$ at *t*-th iteration is

(5) $${\vec{w}}^{\left(t+1\right)}=\arg\min_{\vec{w}}\left\{{\vec{g}}^{\left(1:t\right)}\cdot \vec{w}+\sigma^{\left(1:t\right)}{∥\vec{w}-{\vec{w}}^{\left(s\right)}∥}_{2}^{2}+\lambda_{1}{∥\vec{w}∥}_{1}+\frac{1}{2}\lambda_{2}{∥\vec{w}∥}_{2}^{2}\right\},$$

where σ is defined as the learning-rate schedule such that $\sigma^{\left(1:t\right)}=1/\eta^{\left(t\right)}$, $\lambda_{1}$ and $\lambda_{2}$ are hyperparameters, and ${\vec{g}}^{\left(1:t\right)}$ is the sum of gradient up to the *t*-th iteration.

The FTRL model adopts per-coordinate learning rates instead of the global learning rate. This approach is quite suitable for the logistic regression problem based on similarity vectors. The coordinates, or dimensions, of the similarity vector, are relatively independent. Therefore, it is more reasonable to use per-coordinate learning rates. In FTRL, the formula for updating the learning rate in dimension *i* at *t*-th iteration is:

(6) $$\eta_{i}^{\left(t\right)}=\frac{\alpha }{\beta +\sqrt{\sum_{s=1}^{t}(g_{i}^{\left(s\right)}{)}^{2}}},$$

where α and β are hyperparameters.

We develop the *FTRL Trainer* component to train the FTRL model. It generates the training set first. The training set is composed of instance pairs’ similarity vectors, as well as their similarity scores. The *FTRL Trainer* will first apply the average function on similarity vectors to obtain initial similarity scores. Then, it will select *m* instance pairs in which the initial similarity scores are higher than a certain threshold and *m* ones in which the initial similarity scores are lower than the threshold. These 2m instance pairs will be scored by users. The similarity scores of matched pairs are considered to be 1, while others are assigned 0.

After generating the training set, the *FTRL Trainer* component trains the FTRL model according to the hyperparameters in the configuration file. The trained model is stored in HDFS so that it can be re-adopted.

FTRLIM is designed with a user-feedback mechanism that allows users to correct the matching results manually. The corrected results will be accepted by *FTRL Trainer* to adjust the parameters of the FTRL model. Users are able to choose a batch of candidate instance pairs and correct the similarity scores, or pick up a certain pair to correct. When updating the FTRL model, since the number of unmatched pairs is much greater than the number of actually matched pairs, the unmatched pairs are subsampled with probability *p* to avoid the sample imbalance problem. The probability can be configured by the user.

### 4.4. Matcher

All candidate pairs will obtain their final similarity scores in this component. This component loads a trained FTRL model and predicts similarity scores with Equation (3). The similarity scores are in the interval [0,1]. As defined in Section 3.2, only instance pairs in which the scores are larger than the manually set threshold θ are possible to be matched. In our experiments, we set θ=0.5 for candidate pairs and θ=0.4 for unique pairs to make unique pairs more likely to be matched. The *Matcher* component selects only the one-to-one matched pairs as the final matching results. Before being sent to the FTRL model, elements of similarity vectors are unified from [0,1] to [−1,1] to satisfy the symmetry of Equation (3).

### 4.5. Configuration

FTRLIM allows users to customize their own FTRLIM framework using configuration files. Users are able to set the attributes for index generation, the properties and relations for comparison, the hyperparameters for the FTRL model, and many other detailed parameters.

## 5. Evaluation

### 5.1. Datasets

#### 5.1.1. Benchmark Datasets

To be compared with other frameworks [12,14,20,22,23,36,48,62,63,64,65,66], we report the experiment results on three benchmark datasets. We choose the A-R-S benchmark from OAEI 2009 and the PR benchmark from OAEI 2010 to test FTRLIM. Besides, since FTRLIM participated in the SPIMBENCH Track at OAEI 2019, the evaluation results are also reported.

The A-R-S benchmark includes three real-world datasets named eprints, rexa, and dblp. These three datasets contain instances from the domain of scientific publications. IM frameworks are required to match instances in which the class is ‘document’ or ‘person’. We choose the larger two datasets, i.e., the rexa dataset and the dblp dataset, to conduct experiments. Since our framework gives the one-to-one matched pairs as results, we select 1308 one-to-one matched pairs as reference matching from the 1540 matched pairs given by the OAEI gold standard. The PR benchmark includes three datasets: Person1, Person2, and Restaurant. Among them, Person1 and Person2 are synthetic datasets, while the data of Restaurant comes from two different real-world data sources. We choose the real-world dataset Restaurant to evaluate our framework. The SPIMBENCH benchmark is composed of two datasets with different scales. The SANDBOX dataset has a smaller scale and has a gold standard, while the MAINBOX has a larger scale but the gold standard is not accessible. IM frameworks are required to determine instances that refer to the same real-world ’Creative Work’ in both datasets, respectively. The statistics of these benchmark datasets are shown in Table 5, where the hash symbol (#) means ’the number of’, the same below.

#### 5.1.2. Constructed Datasets

In addition to the benchmark datasets, we construct three data collections based on the knowledge graph provide by the PermID project. PermID is a project provided by Refinitiv to identify entities in the financial field, which provides unique references for data items, including organizations, funds, and individuals. It aims to help people in the field deal with the problems caused by non-standard data. These three data collections are with different scales and levels of heterogeneity to conduct a more comprehensive evaluation: (i) To verify the effectiveness of blocking algorithms, the number of instances both in source and target knowledge graphs should not be too large. Even if there are 5000 instances in both graphs, the total number of instance pairs will be larger than 5000${}^{2}$ = 25,000,000, which could be a difficulty for comparative frameworks without the blocking step. (ii) To explore the matching quality requires datasets that contain knowledge graphs with various and relatively significant information differences. (iii) To evaluate the scalability and matching efficiency of IM frameworks requires sufficiently large-scale datasets.

We extract three subgraphs from the PermID knowledge graph as three source graphs. After preprocessing, we apply the domain-independent instance matching benchmark generator, Lance [67], to generate target graphs for them. According to the number of instances they have, the three source graphs together with their target graphs are divided into 3 data collections: PermID-5k, PermID-20k, and PermID-L, which contains 5 thousand, 20 thousand and a larger number (more than 20 thousand) of instances, respectively (code: https://github.com/TOJSSE-iData/permid-lance, accessed on 12 May 2021). The data extracted from PermID is stored in RDF format, as is the data processed by the Lance framework.

##### PermID-5k

The PermID-5k data collection contains one source knowledge graph and one target knowledge graph. We extract the knowledge in the PermID project of approximately 5000 exchange-listed companies in the United States to form the source knowledge graph. The extracted knowledge includes company name, headquarters address, official website URL, and management personnel. When applying the Lance framework to generate the target knowledge graph, we use the value-based transformation and structure-based transformation [67] to simulate the difference in the construction of different knowledge graphs in the real world. For the company name, person name, and address, we believe that the main reasons for the difference are the spelling error, and the lack of knowledge, while, for the official website URL, we believe that the difference is mainly due to the lack of knowledge. The statistics of this dataset are shown in Table 6.

##### PermID-20k

There is one source knowledge graph and five different target knowledge graphs in this data collection. The source knowledge graph has 148,342 triples of 21,342 instances. Each of the five target knowledge graphs has 20,518 instances, but the numbers of triples are various. These knowledge graphs involve various aspects of knowledge of exchange-listed companies in different countries, including company name, country, headquarters address, official website URL, and management personnel. We simulate the possible value and structure problems that may exist in the real-world data, including the spelling error, the recording error and the lack of knowledge. When generating target knowledge graphs, we believe that the difference is mainly because of the recording error for the country where the company belongs to. The idea of generating other aspects of knowledge is similar to PermID-5k’s. We generate five target graphs by changing the proportion of value-based transformation and structure-based transformation in the Lance configuration, which is shown in Table 7. The source graph and any one of the five target graphs could be used as an independent dataset to evaluate the matching quality of IM frameworks. Among the five pairs of graphs, the target graph and source graph in PermID-20k-A are the most similar, while the target graph and source graph in PermID-20k-E are the most different.

##### PermID-L

The PermID-L data collection is constructed with the knowledge graph that contains approximately 600,000 companies from different countries. The knowledge includes company name, country, headquarters address, and management personnel. The construction of the target graph is similar to PermID-20k. We firstly generate the target graph based on the extracted source graph. Then, we sample instances together with their knowledge from these two graphs to construct graphs with various scales. The PermID-L collection contains 5 pairs of source-target knowledge graphs, i.e., 5 datasets, in total. The statistical results are shown in Table 8.

### 5.2. Evaluation Settings

Three groups of experiments are designed to evaluate the following hypothesis questions:

- Whether the MultiObJ blocking algorithm enables the instance matching for large-scale knowledge graphs by reducing the number of candidate pairs with only a slight impact on the matching quality?
- Would the FTRLIM framework achieve higher matching quality compared with conventional frameworks?
- Would the FTRLIM framework has excellent scalability and matching efficiency compared with the state-of-art frameworks?

The details of the experiments are illustrated in this section.

To evaluate the effectiveness of the MultiObJ blocking algorithm, we conducted comparative experiments on the Restaurant dataset, the rexa-dblp dataset, and the PermID-5k data collection. The Restaurant dataset is a small and simple real-world dataset, while the rexa-dblp dataset is a large-scale and heterogeneous dataset. The PermID-5k data collection contains knowledge graphs of the middle scale. Hence, the comparison evaluates the proposed blocking algorithm under different situation. We choose the recently proposed blocking algorithm by RiMOM-IM [18] (we call it RIMOM-IM-Blk, the same as below) as the baseline to present MultiObJ’s ability to select fewer candidate pairs. We reproduced the RIMOM-IM blocking algorithm because their open-source code is not available now. Besides, versions of FTRLIM with and without the blocking algorithm are compared on PermID-5k to test the affect of MultiObJ on the matching quality. We do not choose a larger data collection, since it will take an unpredictably long time, as well as large memory for the non-blocking version of FTRLIM to obtain the matching results.

To evaluate the matching quality of FTRLIM, we provide historical evaluation results and extended comparative experiment results. Firstly, we provide the results of the SPIMBENCH Track at OAEI 2019. The evaluation results indicate that FTRLIM is able to obtain higher matching quality than the state-of-the-art frameworks. Secondly, we report the evaluation results on the benchmark datasets and the PermID-20k data collection to evaluate the capability of FTRLIM more comprehensively. We choose more than ten frameworks as comparative candidates, which includes both OAEI participants and other state-of-the-art frameworks.

To evaluate the scalability and efficiency of FTRLIM, we conduct experiments on the PermID-L collection. We change both the scale of the cluster and the scale of datasets in our experiment. As a comparison, we duplicated the open-source code of the AML [34] project to process the same datasets. We have also tried to find the code of other excellent frameworks but have not found them yet.

For the evaluation metrics, we calculate the pair completeness (PC) and the pair reduction rate (RR) following the previous works [18,60], which reflect the effectiveness of the blocking algorithm. The metric PC indicates how blocking algorithms affect the matching quality, while the metric RR shows the ability of the block algorithms to reduce candidate pairs. Recall that $I_{S}$ and $I_{T}$ are the sets of instances contained in the source knowledge graph *S* and the target knowledge graph *T*, respectively. Let $B_{S,T}$ be the set of matchable pairs found by the blocking algorithm, $C_{S,T}$ be the set of candidate pairs generated by the blocking algorithm, and $M_{S,T}$ be the set of actually matched pairs between *S* and *T*. Ten PC and RR are defined as:

$$PC=\frac{|B_{S,T}|}{|M_{S,T}|}$$

$$RR=1-\frac{|C_{S,T}|}{|I_{S}\times I_{T}|}.$$

We also adopt the precision, recall, F1-score and time cost as the metrics. Let $P_{framework}$ be the set of matched pairs found by the framework, $TP_{framework}$ be the set of actually matched pairs in $P_{framework}$. The precision, recall, and F1-score are defined as:

$$\mathrm{precision}=\frac{|TP_{framework}|}{|P_{framework}|}$$

$$\mathrm{recall}=\frac{|TP_{framework}|}{|M_{S,T}|}$$

$$F1\text{-}\mathrm{score}=2*\frac{\mathrm{precision}*\mathrm{recall}}{\mathrm{precision}+\mathrm{recall}}.$$

FTRLIM uses Apache Hive as the data warehouse, and all constructed data collections are imported into Hive for storage before all experiments. Predicates in all the mentioned datasets are aligned to fit Assumption 1 proposed in Section 3.2. FTRLIM includes the operation of manually labeling, and the time of manual work is uncertain. Therefore, all the statistics on time cost in our experiments do not include the time-consuming of manual operations. When reporting the experiment results, we use **bold** font to mark the best results over each metric. All the experiments are conducted on a Spark cluster with 48 cores and 64 G RAM. The basic frequency of each core is 2.5 GHz.

### 5.3. Evaluation of the MultiObJ Blocking Algorithm

When experimenting with the Restaurant dataset, we use the predicate *isInCity* as *EP* and *hasName* as *DP* to block the instances. We use the predicate *hasType* as *EP*, *hasName* and *hasLabel* as *DP* to complete the blocking process on the rexa-dblp dataset. Since the knowledge graphs in rexa-dblp are heterogeneous, the blocking algorithm of RIMOM-IM will do a Cartesian product between sets of instances with the same type in different graphs to generate candidate pairs. In this case, the algorithm will generate a huge amount of candidates pairs. Therefore, we also report the results of the RIMOM-IM-Blk algorithm after excluding instance type information for comparison. On PermID-5k, we regard the predicate *hasCompanyName* as *DP* to block instances. The experiment results on these two benchmark datasets are summarized in Table 9.

On the Restaurant dataset, both the MultiObJ blocking algorithm and the RIMOM-IM-Blk algorithm achieve the PC of 1, but the RR of MultiObJ is 28% higher than that of RIMOM-IM-Blk. On the rexa-dblp dataset, MultiObJ finds 14 fewer matchable pairs than RIMOM-IM-Blk and has about 1% lower PC than the latter algorithm. However, the number of candidate pairs generated by the proposed method is only 1/819 of RIMOM-IM-Blk’s. When the type information is excluded, the number of instance pairs generated by RIMOM-IM-Blk is still about 23 times that of MultiObJ, while PC is about 7% lower. On the PermID-5k data collection, although RIMOM-IM-Blk has 3% higher PC than MultiObJ has, its number of candidate pairs is 6 times that of MultiObJ. As we analyzed in Section 2, MultiObJ can generate fewer candidate pairs due to the consideration of the interaction between different predicates.

When conducting experiments on the PermID-5k data collection, we also compare the matching quality between the version of FTRLIM with and without the MultiObJ blocking algorithm. We use the company’s name, headquarters address, official website URL, and employee name to generate similarity vectors. For the FTRL model, we set $\lambda_{1}=0.5,\lambda_{2}=1,\alpha =0.05,\beta =1$. The training set of the FTRL model is generated using 200 manually labeled samples. The experiment results are shown in Table 10.

The FTRLIM framework achieves the precision of 0.984 and the recall of 0.958 in this experiment, while the version without *Blocker* achieves the precision of 0.977, and the recall of 0.999. The PC is 0.960, which means that the blocking algorithm proposed in this paper will discard some pairs of instances with low similarity, even if they may refer to the same entity in the real world. However, the MultiObJ blocking algorithm still has the ability to achieve a high F1-score since its precision is high. Compared with the version without *Blocker*, the number of candidate instance pairs is drastically reduced and RR reaches 0.999, which leads to a significant improvement in the matching efficiency of the FTRLIM framework. The experiment results prove that the MultiObJ blocking algorithm can greatly reduce the matching time while ensuring that the matching results are almost unaffected.

### 5.4. Evaluation of the Matching Quality

FTRLIM has taken part in the SPIMBENCH Track at OAEI 2019, and the results of the track are shown in Table 11. The track has evaluated multiple frameworks on a specific platform, and we have made appropriate adjustments to FTRLIM to meet the requirements of the platform. Nevertheless, the results of the competition can reflect the excellent performance of the FTRLIM framework to a certain extent. In SANDBOX, we got the highest F1-score with the least time cost and achieved 1.00 on the recall. In the larger MAINBOX, we also got the almost highest F1-score with the least time cost, and the recall is as high as 0.998. The evaluation results prove that our framework can obtain a high F1-score and has a low time complexity.

In addition to OAEI 2019, we compare the FTRLIM framework with the OAEI participants [12,48,62,63,64,65,66] and other state-of-the-art frameworks [14,17,20,23,36] on the Restaurant dataset and the rexa-dblp dataset. We adopt the same configurations as described in Section 5.3 to construct indexes for instances. For the Restaurant dataset, we generate the similarity vectors with the restaurant’s name, phone number, and street information. We only select 30 labeled samples to train the FTRL model since the Restaurant dataset is relatively small. The hyperparameters of FTRL are set as $\lambda_{1}=0,\lambda_{2}=0.5,\alpha =0.02,\beta =1$. For the rexa-dblp dataset, we leverage the label of document, the name of person, and the relation between person and document to generate the similarity vectors. We select 300 labeled samples to train the FTRL model, considering that the rexa-dblp is a large-scale real-world dataset. The hyperparameters of FTRL are set as $\lambda_{1}=0.5,\lambda_{2}=1,\alpha =0.1,\beta =1$. The experiment results and the comparison with other frameworks are reported in Table 12. We round the results to the nearest hundredth like the results given by OAEI.

FTRLIM achieves very competitive results on the two real-world datasets. In the relatively simple dataset Restaurant, FTRLIM obtains the F1-score of almost 1. Actually, there is only one matched pair that FTRLIM has not found. In the more complex dataset rexa-dblp, FTRLIM also obtains the F1-score that is almost the same as the best results. We notice that some matchable pairs are lost during the blocking process, which slightly affects the final matching results. FTRLIM does not exceed VDLS’s best results [20] on both of the two datasets. However, from Reference [20], we find that, even on small datasets, such as eprints-rexa, VDLS takes a long time to complete the matching task. FTRLIM makes a trade-off between the matching quality and matching efficiency, and it has the ability to match large-scale knowledge graphs more efficiently.

**Figure 2:**
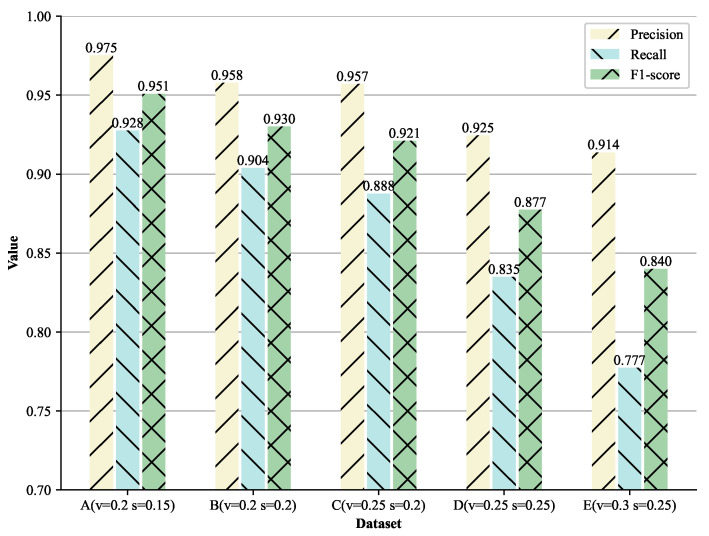
Evaluation Results of FTRLIM on PermID-20k. The level of heterogeneity increases from A to E. The percentage of the transformation is marked in parentheses, and *v* indicates the value-based transformation, while *s* indicates the structure-based transformation. See description of PermID-20k in Section 5.1.2 for details.

We also explore the effect of different levels of heterogeneity between knowledge graphs on the PermID-20k data collection. In terms of the FTRLIM’s configuration, we regard the predicate *hasCountry* as *EP* and the predicate *hasCompanyName* as *DP* to construct instance indexes, and we use the company name, headquarters address, official website URL, and employee name for generating the similarity vectors. For the initialization of the FTRL model, we set $\lambda_{1}=0,\lambda_{2}=0.5,\alpha =0.05,\beta =0.5$. The training set of the FTRL model is generated using 200 labeled samples. The results are shown in Figure 2.

Comparing the experiment results on PermID-20k-B and PermID-20k-C (or PermID-20k-D and PermID-20k-E), it can be seen that the difference in data value will have a greater impact on the *recall* than on the *precision*. We believe that the difference in data value results in a greater impact on the index construction results. For example, when *KIC* strategy is selected, some uncommon words that occur due to the spelling error will be selected as keywords, causing the algorithm to generate wrong indexes for instances. Judging from the overall experiment results, FTRLIM is better at coping with problems of data value, such as the spelling error and the recording error, compared with problems of data structure, such as the lack of knowledge. We argue that the lack of knowledge is a more severe problem because the helpful information for matching instances may be missing with the knowledge.

### 5.5. Evaluation of the Scalability and Efficiency

The scalability and efficiency of the distributed architecture adopted by FTRLIM are verified in two groups of experiments on the PermID-L data collection.

In the first group of experiments, we verify the scalability of our framework with the PermID-L-150k dataset. The FTRLIM framework is deployed on a distributed Spark cluster. By adding or removing cores in the cluster, we conduct multiple experiments. We report the time cost to demonstrate how the processing capacity of the FTRLIM framework changes with the number of Spark cores.

**Figure 3:**
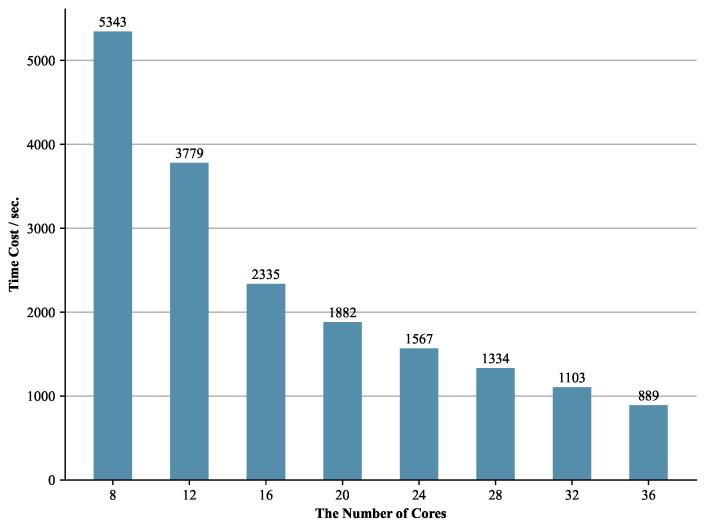
The time cost of FTRLIM with changing the number of Spark cores on the PermID-L-150k dataset.

The results of this group of experiment are shown in Figure 3. The time cost of the framework to complete the matching decreases when the number of cores in the cluster increases. Comparing the time cost when the number of cores is 8 and 36, it can be found that the matching time has been reduced to 1/6, while the number of cores has only increased to 4.5 times. This phenomenon occurs since when the number of cores is small, although all the cores have been already performing computing tasks, there are still tasks waiting to be processed. In a more general scenario, if the number of cores increases to *n* times, the matching time should be no less than 1/n of the original because of the existence of communication cost. The experiment results indicate that when the total amount of data is fixed, adding cores to the cluster will improve the matching efficiency, which demonstrates the excellent scalability of FTRLIM. The results also support the discussion later in Section 6.

Benefiting from the scalability, FTRLIM is able to match knowledge graphs with different scales. The second group of experiment uses a configuration similar to Section 5.4. The AML framework [34] is selected to process the same data as a control.

The results are shown in Table 13. The FTRLIM framework can overcome the challenges brought by the growth of data scale. The time cost of FTRLIM is linearly positively related to data size. Even though the data size increases to 15 times, the time cost increases only by 2 times approximately. However, for the AML framework, we have not obtained valid results on the PermID-L-300k dataset or larger datasets due to the long time cost and high memory requirement. The results show that the distributed FTRLIM framework can process data with different sizes relatively stably and demonstrate the efficiency of FTRLIM in the large-scale data processing.

## 6. Discussion

In this section, we discuss the time complexity of each component of FTRLIM, focusing on the analysis of the MultiObJ blocking algorithm. We also explain how the time performance of the FTRLIM framework changes when the number of Spark cores in the distributed cluster changes.

For the source and target knowledge graph to be matched, we assume that the number of instances in each graph is *N*. Instances from the two graphs will form unique and candidate instance pairs via the MultiObJ blocking algorithm in *Blocker*. The inputs of MultiObJ are the source knowledge graph *S*, the target knowledge graph *T*, and an ordered list of predicates *P*. The algorithm generates the candidate pair set *C* and the unique pair set *U*.

The first phase of the MultiObJ blocking algorithm is *Initialization*. In this phase, the algorithm first creates and initializes required data structures C,U,K, and *B*. For each instance, the algorithm records the initial index $k_{init}$ of the instance in the table *K* and records the instance corresponding to the initial index in the table *B*. In this phase, the algorithm needs to traverse all instances, so the time complexity is O(N).

The second phase of MultiObJ is *Indexing*, including *Explicit Indexing*, *Unique Pair Generation*, and *Index Inference*. The algorithm will go through the loop and construct indexes for instances according to each predicate in the input *P* in turn. Let *l* be the number of loops the algorithm has reached, where *l* ranges from 1 to |P|. As mentioned in Section 4.2, objects corresponding to the predicate may be missing, and the degree of missing objects is described with the completeness rate. Let the average completeness rate over all predicates in *P* of the source and target knowledge graph be δ, in which the range is [0,1]. For an instance in the *l*-th loop, we use $E_{e}^{l}$ and $E_{r}^{l}$ to represent the expectation of the number of indexes obtained in the *Explicit Indexing* phase and in the *Indexing Reasoning* phase, respectively. And we use $E_{g}^{l}$ to indicate the expectation of the number of indexes obtained during the entire *Indexing* phase. $E_{e}^{0}$ and $E_{g}^{0}$ are assigned 1 since the only index of an instance before the *Indexing* phase is the $k_{init}$. The number of distinct indexes is the same as the number of blocks. The expectation of it in the *l*-th loop is denoted as $E_{u}^{l}$. In the following analysis, we first give the time complexity of each phase represented by these expectations and then give the final time complexity representation by deducing the relationship between them.

At the phase of *Explicit indexing*, line 8 of Algorithm 1 divides all instances into four sets, $S_{v},S_{n},T_{v}$, and $T_{n}$, depending on whether corresponding objects of the instance under predicate *p* are missing. The subscript *v* indicates the set contains instances in which the corresponding objects are not missing, while the subscript *n* indicates the opposite condition. Therefore, for the number of elements in each set, we have $|S_{v}\left|=\right|T_{v}|=\delta N$ and $|S_{n}\left|=\right|T_{n}|=\left(1-\delta \right)N$. This step needs to be completed by traversing all the instances, so the time complexity is O(N). The number of executions of the loop at line 9 is δN. The algorithm extracts features of objects at line 10, and the time complexity of this step is O(1), regardless of the index construction strategy. For the *FIC* strategy, the object feature $F_{p,i}$ of an instance is exactly the object under *p*, so the complexity is O(1). For the *KIC* strategy, we need to count the word frequency on all objects under *p* in the two knowledge graphs and store the results in HDFS. The time complexity of the statistics is O(N). However, in practice, the statistics should be carried out in preprocessing. If we assume that the average length of each word is 8 letters, storing a letter requires 2 bytes, storing the word frequency requires 4 bytes, then storing the word frequency of $10^{6}$ words only requires about 20MB. For Spark cores, the time-consuming of reading such word frequency tables from HDFS is negligible. Therefore, after the word frequency table is constructed, the time complexity of identifying the corresponding word frequency could be O(1). In Algorithm 2, experience has shown that the number of construction results generally does not exceed 5, so it can be regarded as a constant, which means the time complexity of using *KIC* strategy to generate $F_{p,i}$ is also O(1). For each instance, the number of $F_{p,i}$ in each loop is denoted as *v*. The line 11 of MultiObJ constructs $E_{e}^{l}$ indexes for each instance, in which the time complexity is $O(E_{e}^{l})$. And we have that

(7) $$E_{e}^{l}=v\cdot E_{g}^{l-1}.$$

The update of the inverted index table $B_{p}$ needs to traverse the constructed indexes, so the time complexity is also $O(E_{e}^{l})$. Therefore, the time complexity of the *Explicit Indexing* phase is $O\left(N\right)+O\left(\delta N\cdot E_{e}^{l}\right)$.

At the phase of *Unique Pair Generation*, MultiObJ needs to traverse $B_{p}$ to identify unique instance pairs. Keys of dictionary $B_{p}$ are distinct indexes constructed in *Explicit Indexing* phase, the minimal number of which is 1. The maximum number of keys of $B_{p}$ is $2\delta N\cdot E_{e}^{l}$. This situation occurs when all the indexes constructed in the *Explicit Indexing* phase are different from each other. On average, the number of keys of $B_{p}$ is

(8) $$E_{u}^{l}=\delta N\cdot E_{e}^{l}=\delta vN\cdot E_{g}^{l-1}.$$

Therefore, the time complexity of *Unique Pair Generation* is $O(\delta N\cdot E_{e}^{l})$.

The *Index Inference* phase of MultiObJ infers indexes for instances in which the objects under predicates are missing. In this phase, the algorithm searches for all suitable instance $j\in G_{v}^{\prime }$ for each instance $i\in G_{n}$, where *i* and *j* have the same index in loop l−1 and $G,G^{\prime }\in \left\{S,T\right\},G\neq G^{\prime }$. The indexes of the instance *j* in the *l*-th loop will become a part of the index set of the instance *i*. The other part of the index set is formed by concatenating each index of instance *i* in previous loop and *NULL*. The instance *i* obtains $E_{g}^{l-1}$ indexes in previous loop, and each index corresponds to multiple eligible instances *j*. In loop l−1, instances in a knowledge graph generates $N\cdot E_{g}^{l-1}$ indexes, among which the number of distinct indexes is $E_{u}^{l-1}$. According to Equation (8), the average number of repetitions for each index is $E_{g}^{l-1}/\left(\delta E_{e}^{l-1}\right)$, which is also the eligible *j*’s quantity. The instance *j* obtains $E_{e}^{l}$ indexes in the *l*-th loop, so the number of indexes obtained in the *Index Inference* phase of each instance with missing objects is

(9) $$E_{r}^{l}=E_{g}^{l-1}\cdot \left(1+\frac{E_{g}^{l-1}}{\delta E_{e}^{l-1}}\right)\cdot E_{e}^{l}.$$

There are (1−δ)N instances in $I_{n}$, so the time complexity of the *Index Inference* phase is $O(\left(1-\delta \right)N\cdot E_{r}^{l})$

The relationship between the aforementioned expectations is deduced as follows. In loop *l*, there are $2N\cdot E_{g}^{l}$ indexes in the *Indexing* phase, which consist of $2\delta N\cdot E_{e}^{l}$ indexes constructed in the *Explicit Indexing* phase and $2\left(1-\delta \right)N\cdot E_{r}^{l}$ indexes constructed in the *Index Inference* phase. Therefore,

(10) $$E_{g}^{l}=\delta E_{e}^{l}+\left(1-\delta \right)E_{r}^{l}.$$

According to Equations (7)–(10), the recurrence relation of $E_{g}^{l}$ can be derived as

(11) $$E_{g}^{l}=\delta v\cdot E_{g}^{l-1}+\left(1-\delta \right)v\cdot {\left(E_{g}^{l-1}\right)}^{2}\left(1+\frac{E_{g}^{l-1}}{\delta E_{e}^{l-1}}\right).$$

Recall that $E_{e}^{0}=E_{g}^{0}=1$, then $E_{e}^{1}=v,E_{r}^{1}\approx v,E_{e}^{2}\approx v^{2},E_{r}^{2}\approx v^{3},E_{e}^{3}\approx v^{4},E_{r}^{2}\approx v^{9},\cdots$, and $E_{g}^{l}\approx E_{r}^{l}$. It can be seen that the closer the average predicate completeness rate δ is to 1, the smaller the high-order items in $E_{g}^{l}$ are, and the smaller the algorithm overhead is. When the number of loops *l* reaches 3, since the exponent of *v* in $E_{g}^{l}$ is too high, the influence of the constant *v* on the complexity of the algorithm cannot be ignored. Therefore, it is not recommended to construct indexes with more than 3 predicates. When |P|≤2, $E_{g}^{l},E_{e}^{l},E_{r}^{l}$ could be regard as constants, and the time complexity of *Indexing* phase is O(N).

The final phase of MultiObJ is *Candidate Pair Generation*. The number of keys in dictionary *B* is $E_{u}^{\left|P\right|}$, and the number of instances from different KGs under each key is $N\cdot E_{g}^{\left|P\right|}/E_{u}^{\left|P\right|}$. The time complexity of this phase is $O\left(E_{u}^{\left|P\right|}{\left(N\cdot E_{g}^{\left|P\right|}/E_{u}^{\left|P\right|}\right)}^{2}\right)∼O\left({\left(N\cdot E_{g}^{\left|P\right|}\right)}^{2}/E_{u}^{\left|P\right|}\right)$.

From the above analysis, we can know that the time complexity of MultiObJ is $O\left(N\right)+O\left(N\cdot E_{g}^{\left|P\right|}\right)+O\left({\left(N\cdot E_{g}^{\left|P\right|}\right)}^{2}/E_{u}^{\left|P\right|}\right)$, where $E_{g}^{\left|P\right|}$ is the expectation of the number of indexes for an instance constructed referring to all the predicates in *P*, and $E_{u}^{\left|P\right|}$ is the expectation of the number of distinct indexes among these indexes. MultiObJ leverages the joint of multiple objects’ features to make indexes of instances more discriminative. In this way, the algorithm increases $E_{u}^{\left|P\right|}$ to reduce the high order term $O({\left(N\cdot E_{g}^{\left|P\right|}\right)}^{2}/E_{u}^{\left|P\right|})$ in the time complexity. In particular, the time complexity of MultiObJ is O(N) when |P|≤2.

After all pairs to be matched are generated, the framework will generate a similarity vector for each pair. For all pairs of instances, FTRLIM sequentially compares the similarity of related objects according to the predicates in the specified predicate set $P_{c}$. FTRLIM generates |U|+|C| instance pairs through *Blocker*, the total number of instance pairs does not exceed 2|C| because unique instance pairs are all candidate instance pairs. Since $|P_{c}|$ can be regarded as a constant, and $O\left(|C|\right)\approx O\left({\left(N\cdot E_{g}^{\left|P\right|}\right)}^{2}/E_{u}^{\left|P\right|}\right)$, the time complexity of comparison is $O(|P_{c}\left|(|U\right|+\left|C|)\right)\approx O\left({\left(N\cdot E_{g}^{\left|P\right|}\right)}^{2}/E_{u}^{\left|P\right|}\right)$. The generated similarity vectors will be judged by the FTRL model. Since the process of training the FTRL model is non-distributed and involves manual operations, we do not consider the time-consuming impact of this process on the whole. The trained FTRL model accepts similarity vectors as inputs and calculates similarity scores for them. Finally, the instance pairs with scores higher than the threshold are filtered and deduplicated to become the final matching results. The time cost of these two processes is proportional to the number of instance pairs, and the time complexity is $O({\left(N\cdot E_{g}^{\left|P\right|}\right)}^{2}/E_{u}^{\left|P\right|})$. Thus, from the generation of similarity vectors to the generation of the matching results, the time complexity of the framework is $O({\left(N\cdot E_{g}^{\left|P\right|}\right)}^{2}/E_{u}^{\left|P\right|})$.

In summary, the time complexity of the FTRLIM framework to complete the instance matching task is $O\left(N\right)+O\left(\delta N\cdot E_{g}^{\left|P\right|}\right)+O\left({\left(N\cdot E_{g}^{\left|P\right|}\right)}^{2}/E_{u}^{\left|P\right|}\right)$, where $E_{g}^{\left|P\right|}$ is the expectation of the number of indexes for an instance constructed referring to all the predicates in *P*, and $E_{u}^{\left|P\right|}$ is the expectation of the number of distinct indexes among these indexes. This complexity can be simplified to O(N) when |P|≤2, where *P* is the list of predicates specified for constructing inverted indexes for instances. FTRLIM is deployed on a distributed Spark cluster. One entire matching process will be divided into multiple tasks, which will be completed by Spark cores in a distributed manner. Theoretically, increasing the number of Spark cores can reduce the computation time for matching. The result of the analysis shows that FTRLIM has approximately linear time complexity. When encountering large-scale data that is difficult to handle, increasing the number of Spark cores in the cluster will improve the matching efficiency.

## 7. Conclusions

In this paper, we propose a novel blocking algorithm MultiObJ. It extracts and joints features of objects according to the type of predicates to block instances and select candidate pairs. Then, we design a distributed framework FTRLIM for large-scale instance matching based on the MultiObJ blocking algorithm. It leverages the online logistic regression model FTRL to determine whether two instances refer to the same entity in the real world. The framework is implemented in a distributed architecture. We construct three data collections with different scales and different levels of heterogeneity. We conduct comparative experiments on two real-world datasets and the constructed data collections. The experiment results verify the effectiveness of the MultiObJ blocking algorithm. The results also show that the FTRLIM framework performs high-quality instance matching with high efficiency and excellent scalability than the state-of-the-art frameworks.

The FTRLIM framework focuses more on the attributes of instances to construct indexes and compare instance pairs. In the follow-up work, we will consider integrating more structural information of KGs to perform instance matching. In addition, we also find that the current framework still has a certain dependence on human work, and we will also make an effort to improve the automation of the FTRLIM framework.

## Figures and Tables

**Table 1:** An example of the *Indexing* phase.

Pre-Index	Instance	Feature	Current Index
A	S1	X	A$X
A	S2	Y	A$Y
A	S3	-	A$X, A$NULL
B	S4	X	B$X
A	T1	X	A$X
A	T2	X	A$X
A	T3	-	A$X, A$Y, A$NULL
B	T4	X	B$X

**Table 2:** An example of the blocking results.

Block Key	Candidate Instance Pair
A$X	$\langle S1,T1\rangle ,\langle S1,T2\rangle ,\langle S1,T3\rangle ,\langle S3,T1\rangle ,\langle S3,T2\rangle ,\langle S3,T3\rangle$
A$Y	$\langle S2,T3\rangle$
A$NULL	$\langle S3,T3\rangle$
B$X	$\langle S4,T4\rangle$

**Table 3:** An example of the CombKey results on Restaurant dataset with R=2.

ID	hasName	Word Count	Dense Rank	Features
1-45	manhattan	manhattan: 3 ocean: 2 club: 4 21: 2 café: 1	manhattan(1),	manhattan-club
	ocean club		ocean(1), club(2)	ocean-club
2-45	manhattan		manhattan(1),	manhattan-club
	ocean club		ocean(1), club(2)	ocean-club
1-23	21 club		21(1), club(2)	21-club
2-23	21 club		21(1), club(2)	21-club

**Table 4:** An example of the similarity vector generation.

ID	hasLabel	hasAuthor	Sim1	Sim2	Similarity Vector
1-01	A Demo of Label	A.B, C.D, E.F	0.727	0.500	$\langle 0.727,0.500\rangle$
2-01	Another Demo of Label	A.B, M.N			

**Table 5:** Statistics of the benchmark datasets.

Benchmark	Dataset	# Source	# Target	# Gold Standard
A-R-S	rexa-dblp	14,771	1,615,197	1308
PR	Restaurant	113	752	89
SPIMBENCH	SANDBOX	349	354	299
	MAINBOX	1759	1772	-

**Table 6:** Statistics of the PermID-5k data collection.

Knowledge Graph	# Instance	# Triple
Source	5562	31,614
Target	5416	31,572

**Table 7:** Statistics of the PermID-20k data collection.

Dataset	# Target Triple	Transformation Allocation	
		Value	Structural
PermID-20k-A	120,295	0.2	0.15
PermID-20k-B	119,243	0.2	0.2
PermID-20k-C	119,243	0.25	0.2
PermID-20k-D	118,262	0.25	0.25
PermID-20k-E	118,262	0.3	0.25

**Table 8:** Statistics of the PermID-L data collection.

Dataset	Source		Target	
	# Instance	# Triple	# Instance	# Triple
PermID-L-40k	42,149	244,464	40,718	218,746
PermID-L-80k	83,326	472,458	80,499	453,763
PermID-L-150k	153,424	861,782	148,218	843,274
PermID-L-300k	321,244	1,740,178	309,942	1,679,394
PermID-L-600k	604,432	3,264,378	583,923	3,137,179

**Table 9:** The results of MultiObJ compared with RIMOM-IM-Blk.

Dataset	Method	# Instance Pair	# Candidate Pair	PC	RR
Restaurant	RIMOM-IM-Blk	84,976	24,922	1.000	0.7067
	MultiObJ		1097	1.000	0.9871
rexa-dblp	RIMOM-IM-Blk	23,858,074,887	8,706,076,913	0.998	0.6351
	RIMOM-IM-Blk (without type)		244,137,613	0.920	0.9898
	MultiObJ		10,634,939	0.988	0.9996
PermID-5k	RIMOM-IM-Blk	30,123,792	155,746	0.993	0.9948
	MultiObJ		25,382	0.960	0.9992

**Table 10:** Evaluation results of the MultiObJ on PermID-5k.

Method	Precision	Recall	F1-Score	Time Cost (sec.)
FTRLIM	0.984	0.958	0.971	116
FTRLIM	0.977	0.999	0.988	2679
(without *Blocker*)				

**Table 11:** Evaluation results of the SPIMBENCH Track, OAEI 2019.

Dataset	Method	Precision	Recall	F1-Score	Time Cost (ms)
SANDBOX	LogMap [33]	0.938	0.763	0.841	6919
	AML [34]	0.835	0.896	0.865	6223
	Lily [35]	0.849	1.000	0.919	2032
	FTRLIM	0.854	1.000	0.921	1474
MAINBOX	LogMap	0.893	0.709	0.791	26,920
	AML	0.839	0.884	0.860	39,515
	Lily	0.855	1.000	0.922	3667
	FTRLIM	0.856	0.998	0.921	2155

**Table 12:** Evaluation results of FTRLIM and other methods.

Dataset	Method	Precision	Recall	F1-Score
Restaurant	RIMOM [62]	0.86	0.77	0.81
	ASMOV [12]	0.70	0.70	0.70
	LN2R [64]	0.76	0.75	0.75
	CODI [66]	0.72	0.72	0.72
	ObjectCoref [48]	-	-	0.90
	PARIS [23]	0.95	0.88	0.91
	SERIMI [36]	0.77	0.77	0.77
	LINDA [14]	1.00	0.63	0.77
	BIGMAT(RW) [22]	-	-	0.99
	BIGMAT(APRW)	-	-	1.00
	VDLS(α=0) [20]	-	-	1.00
	VDLS(α=1)	-	-	0.98
	FTRLIM	0.99	0.99	0.99
rexa-dblp	HMATCH(I) [63]	0.42	0.48	0.45
	RIMOM [62]	0.80	0.72	0.76
	FBEM [65]	0.99	0.12	0.21
	VMI [17]	0.71	0.78	0.76
	BIGMAT(RW) [22]	-	-	0.92
	BIGMAT(APRW)	-	-	0.94
	VDLS(α=0) [20]	-	-	0.94
	VDLS(α=1)	-	-	0.97
	FTRLIM	0.94	0.96	0.95

**Table 13:** Evaluation results of changing data size.

Dataset	Method	Precision	Recall	F1-Score	Time Cost (sec.)
PermID-L-40k	AML [34]	0.918	0.819	0.866	875
	FTRLIM	0.959	0.887	0.922	644
PermID-L-80k	AML	0.908	0.804	0.853	1942
	FTRLIM	0.940	0.863	0.900	723
PermID-L-150k	AML	0.903	0.777	0.836	>3600
	FTRLIM	0.936	0.854	0.893	1044
PermID-L-300k	AML	-	-	-	-
	FTRLIM	0.933	0.850	0.890	1445
PermID-L-600k	AML	-	-	-	-
	FTRLIM	0.927	0.833	0.878	2034

## Data Availability

Publicly available dataset PermID-Project was processed in this study to construct the PermID-5k, PermID-20k, and PermID-L data collections. The PermID-Project data can be found here: https://permid.org/ (accessed on 10 November 2019).

## Abbreviations

The following abbreviations are used in this manuscript:

FTRL	Follow-the-Regular-Leader
KG	Knowledge Graph
IM	Instance Matching
